# Disparate Climate Change Health Costs: The Emissions, Vulnerability, and Readiness Nexus

**DOI:** 10.1007/s10393-025-01761-7

**Published:** 2025-09-27

**Authors:** Matan E. Singer, Maya Negev

**Affiliations:** 1 School of Public Health, The Faculty of Social Welfare and Health Sciences, University of Haifa, 199 Abba Khoushy Ave., Mount Carmel, Haifa, Israel; 2 Haifa Center on the Politics of Inequality, University of Haifa, 199 Aba Khoushy Ave., Mount Carmel, 3103301 Haifa, Israel

**Keywords:** climate change, health costs, CO_2_ emissions, vulnerability, mortality

## Abstract

The emissions, vulnerability, and readiness nexus illustrate several interrelated disparities between the Global North and South. We examine these disparities by mapping country-level CO_2_ emissions against climate-related mortality. Global North countries and China are the main contributors to CO_2_ emissions, whereas African countries and India are the most affected. This relationship is further reflected in a strong negative association between readiness for and vulnerability to climate change. The dichotomy between the Global North’s contribution and the Global South’s vulnerability to climate change risks underscores the urgent need for equitable climate policies that address these imbalances and bolster global readiness.

**Supplementary Information:**

The online version contains supplementary material available at 10.1007/s10393-025-01761-7.

Climate change poses the greatest threat to global health, increasing morbidity and mortality worldwide (Atwoli et al., 2021). Changes in temperature, humidity, and precipitation increase the incidence of infectious diseases, including vector-, food-, and water-borne diseases such as malaria, dengue, and cholera (Mahon et al., 2024; Mora et al., 2022; Rohr & Cohen, 2020). Extreme weather events increase mortality, reduce water security, and adversely affect mental health (Berry et al., 2018; IPCC, 2022; Rocque et al., 2021). All regions of the world are at risk, and the populations of both the Global North and Global South are exposed to climate change. However, climate change exhibits extreme disparities within and between countries (Watts et al., 2021).

The seminal work of Patz et al., (2007) and Althor et. al., (2016) highlighted the mismatch between countries disproportionately driving climate change through greenhouse gas (GHG) emissions and those disproportionately affected by it. The Global North, with its high per-capita and overall emissions, contributes significantly to the problem while also benefiting economically and maintaining high levels of readiness. In contrast, the Global South produces low emissions, coupled with relatively low readiness and heightened vulnerability (Watts et al., 2021). However, these studies do not fully account for emission trends past 2010, while recent studies have examined each topic separately. Juxtaposing GHG emissions, a proxy for climate change contribution, and vulnerability to and readiness for climate change is crucial from environmental health, justice, and policy perspectives, aiming to identify the sources of pollution and emissions alongside communities that are disproportionately affected and their ability to cope with the impacts of climate change.

This paper adopts a geospatial approach to examine the interrelated disparities associated with the emissions–vulnerability readiness nexus by visualizing the spatial mismatch between the main contributors to climate change and the countries most affected by it. Our objective is not to draw a direct causal link between emissions and vulnerability. Rather, we aim to update and affirm the observational (negative) relationship between emissions and vulnerability. Achieving this would allow holding contributors accountable and direct mitigation efforts toward the most vulnerable. In doing so, we assume that higher emissions, especially over time, are related to greater economic development, allowing countries to be better prepared for climate-related health risks. We employ comprehensive publicly available databases on national contributions to CO_2_ emissions (EMDGAR, 2024), a proxy for climate change contributions through total GHG emissions. We employ climate-related mortality (GBD) as a proxy for the health-related impact of climate change. Finally, we use the ‘readiness’ and ‘vulnerability’ to climate change indices from the NDGAIN database to account for a country’s level of economic development and ability to cope with climate risks (Supplementary Material 1). Based on the IPCC (2014), vulnerability is the ‘propensity or predisposition of human societies to be negatively impacted by climate hazards,’ while readiness is composed of economic, government, and social indicators of the ability to make effective adaptation actions (NDGAIN).

The estimation and computation of country-level CO_2_ emissions, mortality, and ‘readiness/vulnerability’ are constrained by the availability, quality, and consistency of country-level data on the various indicators that are used to calculate these variables. The main portions of the analysis are based on 2019 data to avoid the effects of the COVID-19 pandemic on emissions and climate-related mortality. A detailed description of the data sources and their limitations is presented in Supplementary Material 1.

The analyses are based on a series of maps and cartograms that allow identifying the main contributors to climate change and those that are most affected by it. Cartograms are a type of map that distorts the geometry (e.g., land area) of the mapped region based on the values of another variable (e.g., CO_2_ emissions or mortality). Accordingly, cartograms allow visualizing the countries that contribute disproportionately to climate change and those that are disproportionately affected by it. The cartograms are complemented by maps that break down the spatial mismatch characterizing the emissions–vulnerability–readiness nexus. All maps and cartograms were created in R 4.3.3 using the ‘cartogram,’ ‘sf,’ and ‘ggplot2’ packages.

Mapping country-level CO_2_ emissions and climate-related deaths show the disproportionally high contribution of Global North countries and China to climate change (Fig. 1a, b) while the Global South is the most affected. China was the greatest emitter of CO_2_ in 2019, followed by the USA, Russia, and India. Additionally, Europe, the Middle East, and North and South Africa also show relatively disproportionately high contributions to CO_2_ emissions. Conversely, Central Africa, Latin America, Southeast Asia, and Oceania show disproportionately low contributions to global CO_2_ emissions.

**Figure 1:**
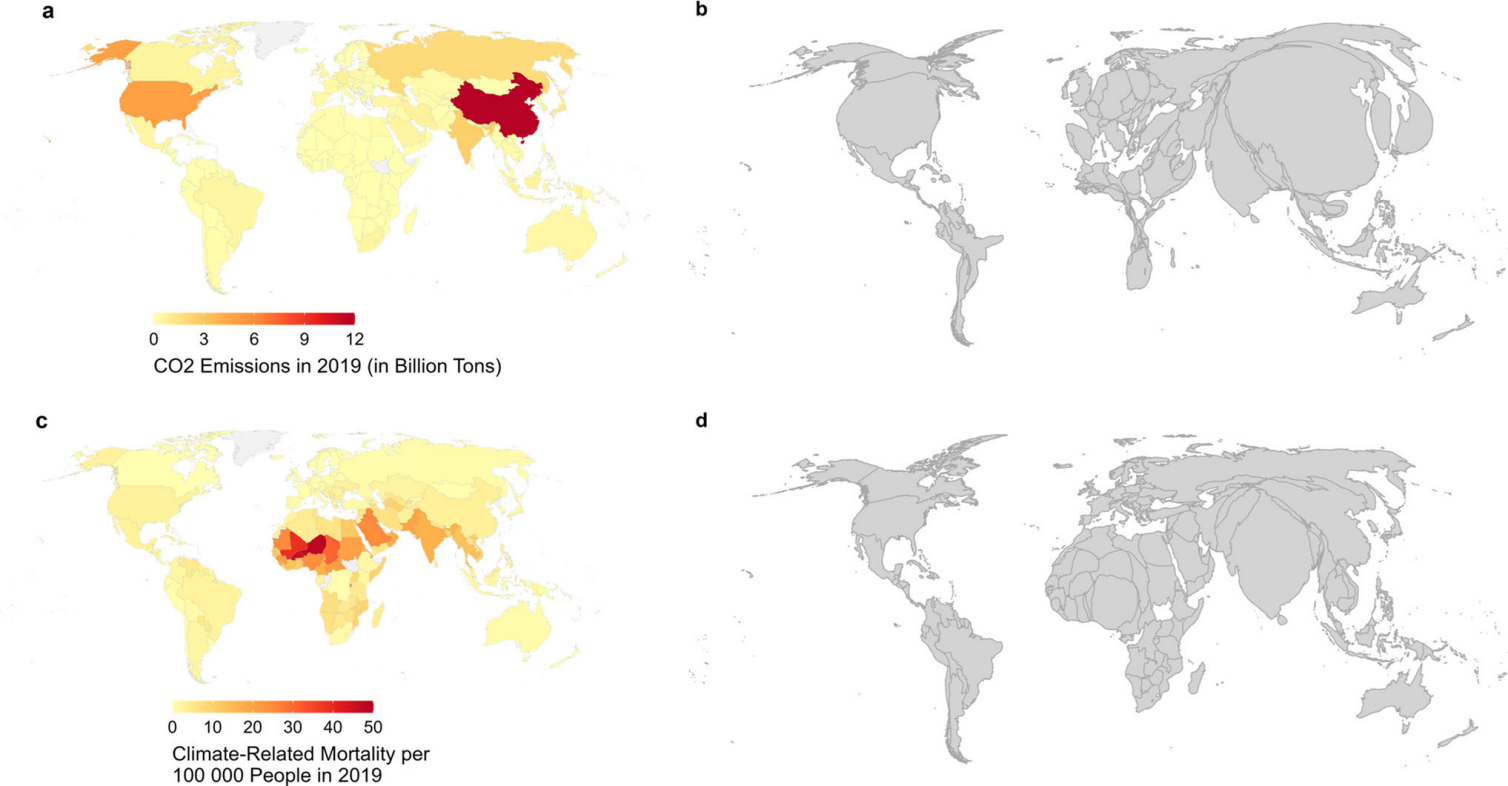
Comparing global CO2 emissions (in Billion Tons) (**a**, **b**) and climate-related mortality (**c**, **d**) by country (2019). b and d are cartograms in which the country size is proportional to emissions (**b**) and mortality (**d**). **a**-**b** China was the greatest emitter of CO2 in 2019, followed by the USA, Russia, and India (**a**). These countries, together with Western Europe and Japan, contribute a disproportionately high amount of global CO2 emissions, while Africa and Latin America show a disproportionately low amount of global CO2 emissions (**b**). **c**-**d** Countries in the northern part of Sub-Saharan Africa, the Middle East, and South East Asia experienced the highest levels of climate-related mortality per 100,000 people in 2019 (**c**). These countries, and especially India, show disproportionately high levels of climate-related mortality (**d**).

Mapping climate-related mortality provides a mirror image of CO_2_ emissions. Country-level climate-related mortality per 100,000 people was calculated by summing two mortality cause and risk factors that are commonly regarded as related to climate change and climate events, malaria, and high temperatures (see Supplementary material 2). Additional mortality risk factors that were considered, including diarrheal diseases, drowning-related death, malnutrition, fire, heat and hot substances, and non-communicable diseases, were not used in the final analysis due to their weaker performance as a climate-related mortality risk factor. Countries in the northern part of Sub-Saharan Africa, the Middle East, and Southeast Asia experienced the highest levels of climate-related mortality per 100,000 people in 2019 (Fig. 1c). These countries, and especially India, show disproportionately high levels of climate-related mortality (Fig. 1d). At the same time, North and Latin America, Europe, and East Asia show a disproportionally low mortality rate from climate-related risks.

Breaking down mortality into the two specific causes and risks shows that mortality rates related to exposure to high temperatures have a wider spread across the northern part of Sub-Saharan Africa, the Middle East, and Southeast Asia (SM 2a). In contrast, malaria-related mortality rates are primarily concentrated in Western and Central Africa (SM 2b).

The visual inverse relationship between CO_2_ emissions and climate-related deaths is further evidenced by the strong negative relationship between each country's readiness for climate change and its vulnerability to climate-related risks (Pearson’s r = -0.71; Fig. 2). North American, Western European, and East Asian countries show high levels of readiness alongside low levels of vulnerability. In contrast, African, Southeast Asian, and Latin American countries, which are more vulnerable to climate change risks, show low readiness levels.

**Figure 2:**
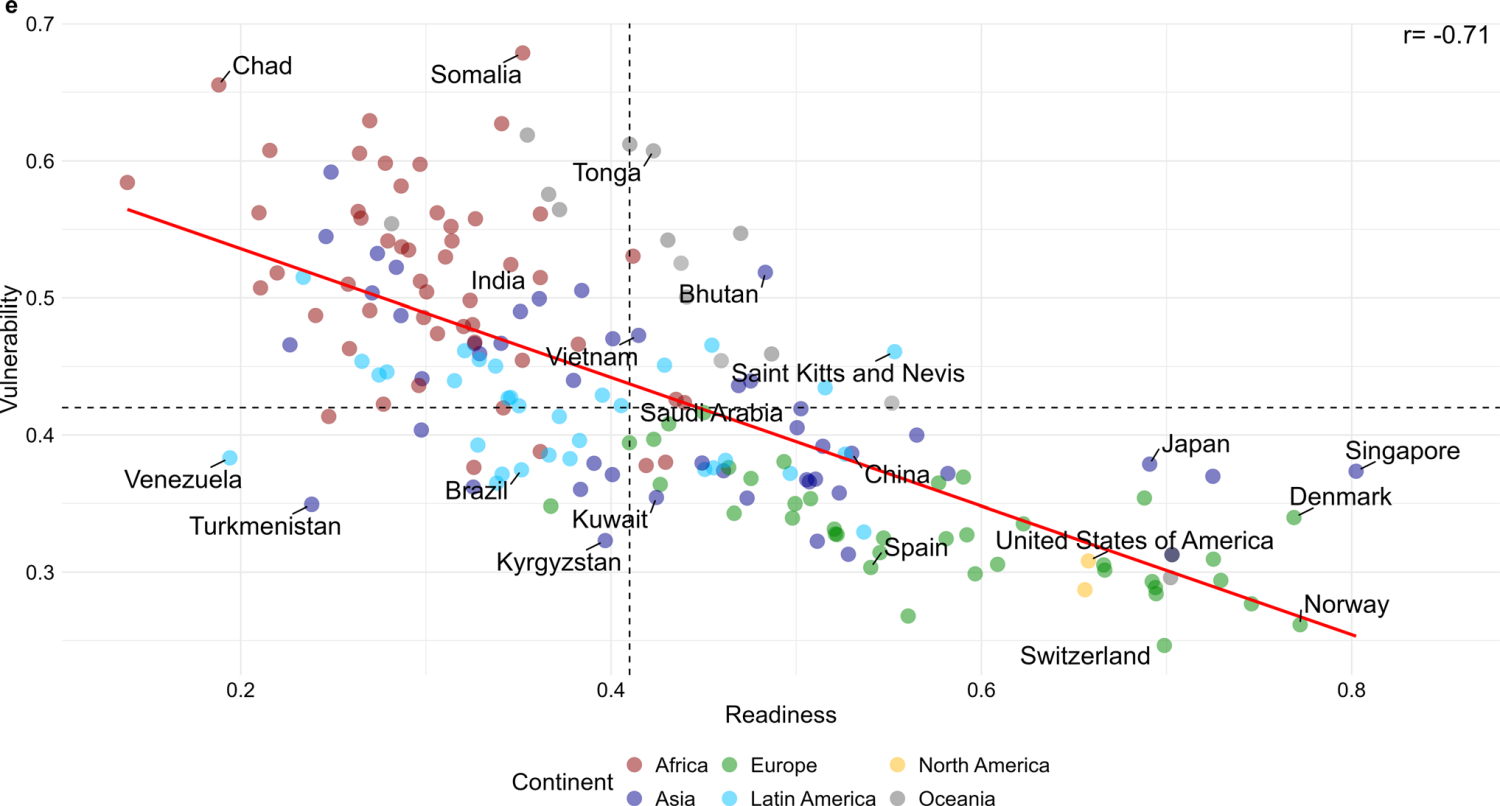
Readiness (x-axis) vs. vulnerability (y-axis) by country (2019). The result is a strong negative association (r = -0.71) between vulnerability to climate change and readiness to cope with climate change at the country level.

Finally, we qualify our analysis using a cartogram mapping CO_2_ emissions per million people in 2019 (Fig. 3a) and a country-level map of the change in total CO_2_ emissions between 1970 and 2019 (Fig. 3b). While China was found to be the greatest emitter of CO_2_ emissions in absolute terms, when population size is accounted for, oil-dominant countries in the Middle East and small countries with a large energy sector, including Trinidad and Tobago and Luxembourg, are the top CO_2_ emitters per million people, with a disproportionate amount of emissions relative to their land size. North America, Australia, and Russia are also in the top decile of CO_2_ emissions per million people, yet their emission levels are relatively proportional to their land area. Sub-Saharan countries dominate the bottom quartile of CO_2_ emissions per million people, whereas the second and third quartiles include countries from Latin America, Africa, and Southeast Asia (Fig. 3a).

**Figure 3:**
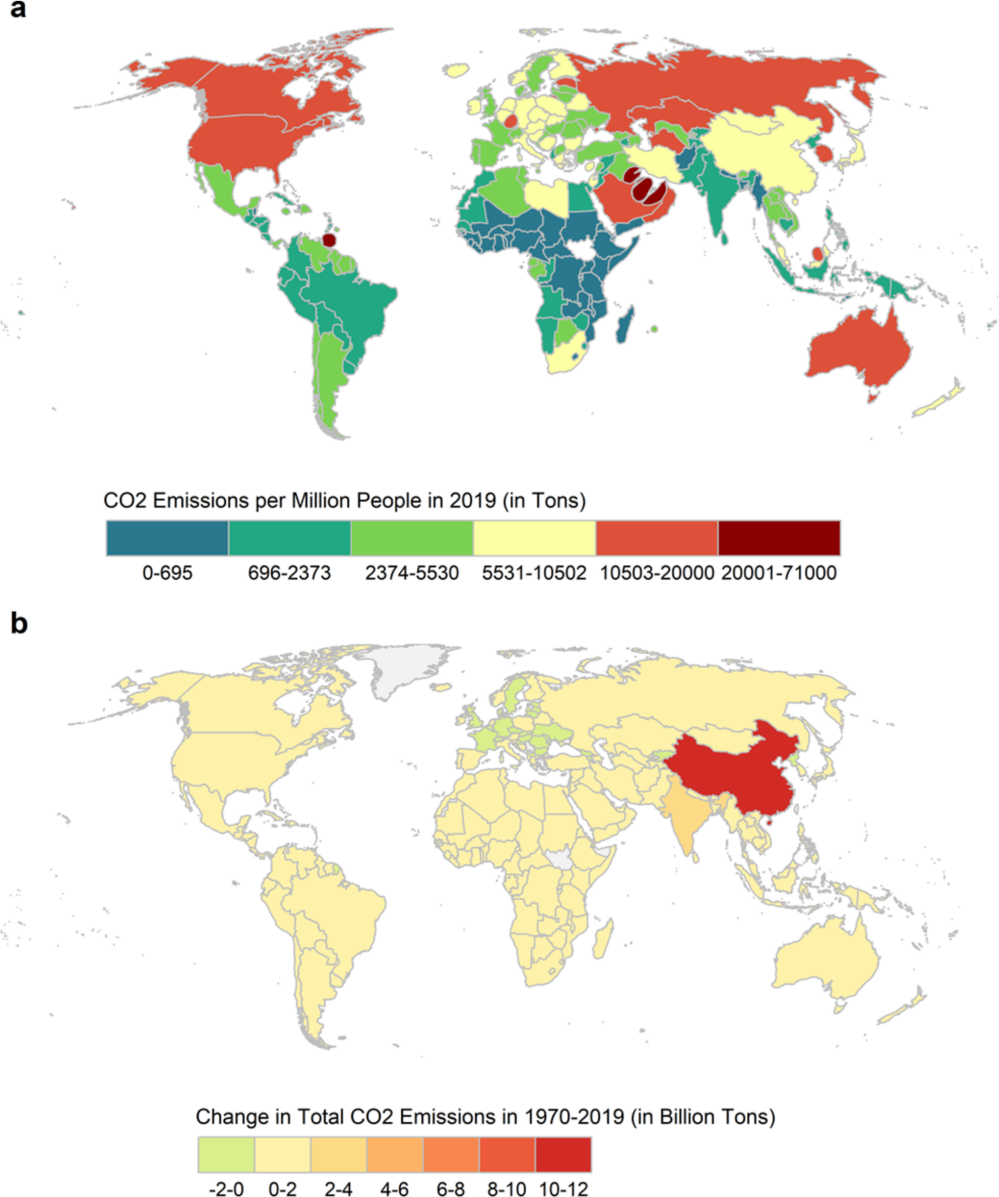
Global CO2 emissions per million people (2019; **a**) and emissions trends (1970–2019; **b**). **a**-**b** Global North countries emit significantly more CO2 per million people than the Global South, with Middle East countries, Luxembourg, and Trinidad and Tobago emitting a disproportionate amount of CO2 relative to their land size (**a**). European countries experienced a slight decline in CO2 emissions between 1970 and 2019, while most other countries experienced a slight increase. India, and especially China, showed the largest increase in CO2 emissions over the studied period (**b**). **a**) is a cartogram in which the country's land size is proportional to emissions. The bottom three levels represent quartiles 1–3, level four represents countries in the 75th-90th percentiles, and levels five and six represent countries in the top decile.

The longitudinal analysis (Fig. 3b) shows that India, and especially China, experienced the largest increase in CO_2_ emissions between 1970 and 2019, particularly since the early 2000s (not shown). During the same period, several European countries experienced a decline in CO_2_ emissions, whereas most other countries experienced a slight increase in emissions (Jones et al., 2023) (Fig. 3b). However, despite the slight decrease in CO_2_ emissions among several European countries, these countries are still in the top two quartiles in terms of emissions per capita. In contrast, despite the increase in China’s emissions over the past five decades, the country’s emissions per million people are only in the 85th percentile of emissions (Fig. 3a).

The contributions of this study are threefold. First, we update the analyses by Patz and Althor to include the decade of 2010–2019, which exhibited significant changes in emissions trends and substantial policy changes among a subset of (mostly European) countries aimed at reducing GHG emissions. Second, we extend Patz and Althor’s analyses by accounting for per-capita emissions. This changes the perspective of countries (namely, China) that contribute the most to climate change. Third, we further extend Patz and Althor’s analyses by evaluating the contribution to climate change and vulnerability to it in the context of structural readiness and vulnerability. Assessing readiness in relation to vulnerability helps to understand the extent of vulnerability and the role of public policy and the national economy in mitigating vulnerability and negative health impacts.

Analyzing the emissions–vulnerability–readiness nexus illustrates at least three interrelated disparities between the Global North and South. First, the industrialized Global North is disproportionately responsible for greenhouse gas (GHG) emissions. Our results echo those of previous studies showing that the top 10% of the world population by income and wealth emits 48% of the total emissions generated by private consumption, investments, and government spending, while the bottom 50% of the world population emits just 12% (Chancel, 2022). Second, GHG emissions tend to show an inverse relationship with vulnerability to climate change at the country level (Althor et al., 2016), with Global South countries facing a disproportionately high burden of climate-related excess morbidity and mortality (Deivanayagam et al., 2023). Third, developing countries have lower levels of readiness to adapt to climate change and often lack the economic, governmental, and social resources to implement adaptation measures that can reduce exposure and vulnerability to climate hazards and consequently reduce excess mortality (Negev et al., 2019; Vicedo-Cabrera et al., 2021). This dichotomy between the Global North and South underscores a complex interplay of factors, such as colonial history, economic globalization, and trade patterns, which contribute to disparities in contributions to climate change, readiness, and vulnerability.

Compared to (Patz et al., 2007), the proportional contributions of Europe and the USA to global CO_2_ emissions have somewhat declined in the past two decades, while that of China has increased considerably. Nonetheless, affluent countries, including European countries, remain among the largest contributors to climate change (Jones et al., 2023). These countries exhibit relatively low vulnerability to climate change risks and high climate readiness, resulting in lower climate-related mortality. In contrast, within the Global South, which contributes only a fraction of global GHG emissions, the population is disproportionately vulnerable to climate risks, in part due to lower climate readiness capacity, and therefore experience elevated rates of climate-related health impacts.

Climate change exacerbates existing health disparities between the Global North and South due to higher vulnerability and lower readiness in the Global South, including poor health services (Negev et al., 2019). However, excess climate-related morbidity and mortality are largely preventable (Ebi et al., 2021) given sufficient economic and political investment. The spatial mismatch identified here and elsewhere provides further evidence that a global effort is needed to develop and implement adaptation and mitigation practices that improve readiness and lower vulnerability in the Global South. Climate mitigation and adaptation, including health co-benefits with an emphasis on vulnerable populations, can close the readiness gap between the Global North and South (Campbell-Lendrum et al., 2023; Roca-Barceló et al., 2024). Specifically, the role of the Global North goes beyond mitigation and national adaptation efforts. Historically large emitters should support Global South countries in implementing national adaptation strategies and developing readiness. The UN Climate Change conferences started recognizing this need with the decision to provide loss and damage funding for vulnerable countries (COP27), new global adaptation targets (COP28), and the setting of a new financial goal to triple the finance to developing countries by 2035. However, these decisions have yet to deliver meaningful resources. Amidst the record-breaking extreme climate events in 2025, there is a need to scale up and accelerate the closing of the readiness gap.

## Supplementary Information

Below is the link to the electronic supplementary material.

Supplementary file1 (DOCX 21 kb)

Supplementary file2 (DOCX 314 kb)
