# Supplementary material for: Disparate Climate Change Health Costs: The Emissions, Vulnerability, and Readiness Nexus
Source: Ecohealth. 2025 Sep 27;23(1):11–7. doi: 10.1007/s10393-025-01761-7 (PMC12932275; doi:10.1007/s10393-025-01761-7)
Supplement: Supplementary file 1 — Supplementary file1 (DOCX 21 kb) [file 10393_2025_1761_MOESM1_ESM.docx]

**Supplementary Material**

This study examines the emissions-vulnerability-readiness nexus at the country level using geospatial tools to visualize the spatial mismatch between the main contributors to climate change and the countries most affected by it. This analysis builds on comprehensive publicly available datasets reporting on GHG, mortality by risk and cause factors, and vulnerability to climate change.

CO_2_ emissions data were obtained from the Emissions Database for Global Atmospheric Research (EDGAR; <https://data.jrc.ec.europa.eu/collection/edgar>). The EDGAR v8.0 Greenhouse Gas Emissions dataset includes emission estimates from 1970-2022 by sector and country for three greenhouse gases (CO_2_, CH_4_, N_2_O) and fluorinated gases. EDGAR calculates emission estimates using publicly available data on human activity that causes GHG emissions and emission factors. The calculated emissions estimates include some degree of uncertainty stemming from data quality issues (e.g., completeness, sampling representativeness, treatment of missing data). A second source of uncertainty relates to emission factors (e.g., aggregation assumptions, and random error bias and variability). Among the three GHG EDGAR reports on (CH_4_, CO_2_, and N_2_H), CO_2_ has the largest emission share and the lowest uncertainty share globally (Solazzo et al., 2021). 2019 data were used to align with the latest mortality data available while avoiding the effects of the COVID-19 pandemic on emissions and mortality. We further take advantage of the longitudinal nature of the data and map change in CO_2_ emissions between 1970 and 2019. This allows for identifying relatively recent trends in GHG emissions that have not been accounted for in previous similar studies (Althor et al., 2016; Jonathan A. Patz et al., 2007).

Climate-related mortality is multifactorial and providing a single estimate of the overall burden is challenging. Nevertheless, the World Health Organization assessed several climate-sensitive death causes (Campbell-Lendrum et al., 2023). Here, we present results regarding two of these causes for which there is available data: malaria and high temperature (as a risk factor for mortality due to drowning, suicide, homicide, transport injuries, cardiovascular diseases, etc.). Notably, most deaths from malaria are not due to climate change. Moreover, future social changes, including socioeconomic conditions, demography, health status, health care access, etc., may reduce mortality from malaria despite climate change. At the same time, most cases of climate change-related mortality are not included in this work due to a lack of international data on climate change as a risk factor. With the dual reservation that analyzing malaria and high temperature is both an underestimate of overall climate mortality and an overestimate of climate-related mortality, we use the two climate-sensitive death causes as a proxy for climate-related mortality.

Mortality data were used as a proxy for vulnerability to climate change. The data were obtained from the Global Burden of Disease (GBD) study (<https://vizhub.healthdata.org/gbd-results/>) provided by the Institute for Health Metrics and Evaluation (IHME). The GBD study is a systematic and comprehensive scientific initiative that seeks to quantify the extent of major diseases, risk factors, and intermediate health outcomes in a standardized manner. This approach allows for consistent comparisons across different populations, time frames, and health problems. 2019 mortality data on deaths related to two risk and cause factors were obtained by country: 1) all mortality cases associated with high-temperature as a risk factor; and 2) deaths from malaria, which is associated with all risk factors. ‘Risk Factor’ estimates refer to attributes or exposures that are causally associated with an increased incidence or prevalence of a cause of death (or injury). The GBD study aims to reduce this uncertainty by reporting the 95^th^ uncertainty interval, determined based on the calculation of 1,000 estimates, alongside lower and upper estimates. However, the GBD-calculated estimates still have some degree of uncertainty associated with them, stemming from data availability and quality issues.

Data on ‘Readiness’ for and ‘Vulnerability’ to climate change in 2019 were obtained from the Notre Dame Global Adaptation Initiative (ND-GAIN; <https://gain.nd.edu/our-work/country-index/download-data/>). The ND-GAIN Readiness score combines economic, governance, and social factors that indicate a country’s ability to convert economic and social investments and institutional governance activities into climate adaptation efforts. Vulnerability to climate change measures the degree to which each country’s food, water, health, ecosystem services, human habitat, and infrastructure sectors are exposed to climate change, sensitive to climate hazards, and their capacity to adapt to climate change. Similar to the CO_2_ emissions and mortality data, the computation of country-level ‘vulnerability’ and ‘readiness’ depends on the availability, quality, and consistency of country-level data on the various indicators that are used to calculate these variables.
