# Supplementary material for: Disparate Climate Change Health Costs: The Emissions, Vulnerability, and Readiness Nexus
Source: Ecohealth. 2025 Sep 27;23(1):11–7. doi: 10.1007/s10393-025-01761-7 (PMC12932275; doi:10.1007/s10393-025-01761-7)
Supplement: Supplementary file 2 — Supplementary file2 (DOCX 314 kb) [file 10393_2025_1761_MOESM2_ESM.docx]

**Supplementary Material File 2 – High temperature-related and Malaria-related Deaths per 100,000**


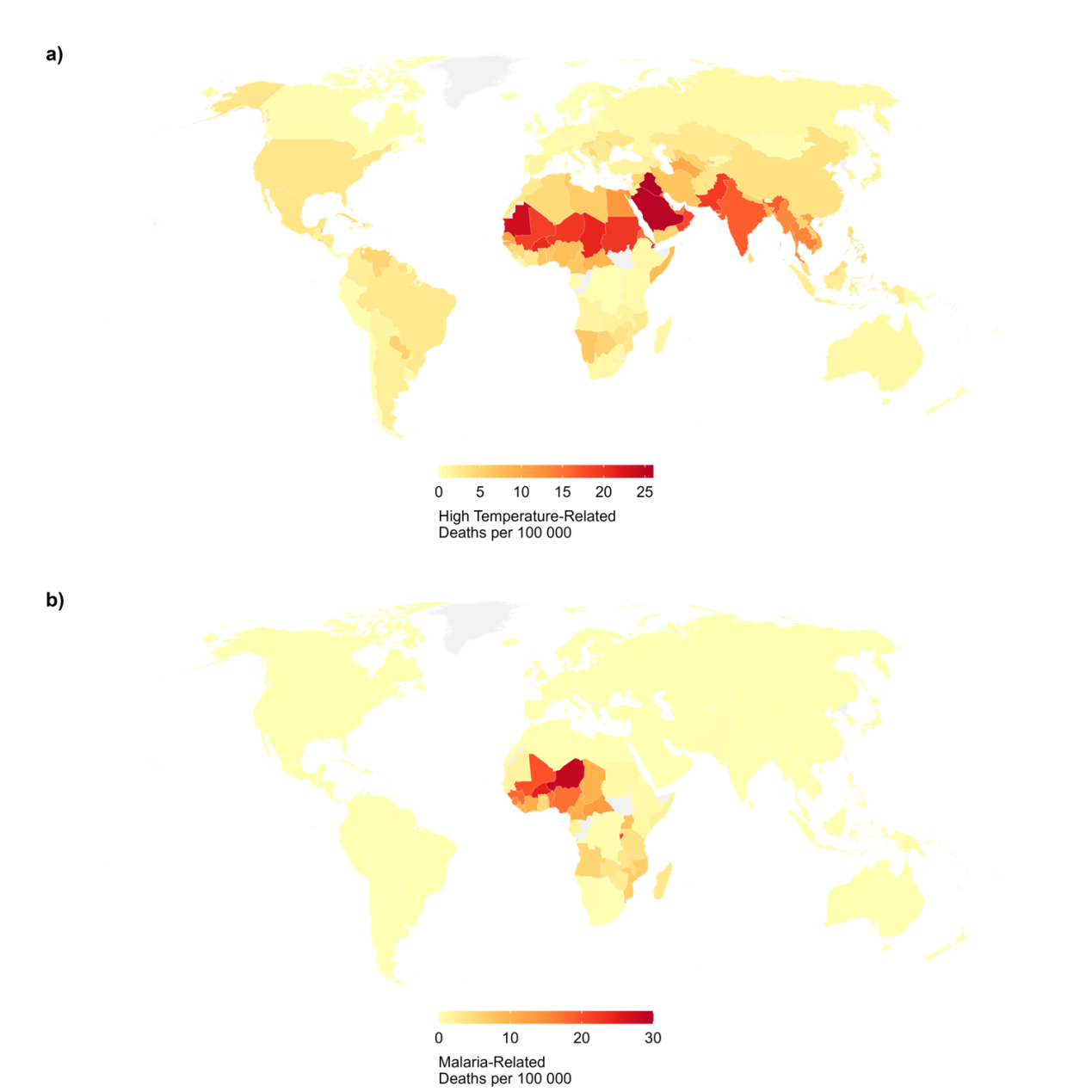


SM 2. Climate-related mortality from high temperatures (a) and malaria (b) as risk factors, in 2019. Country-level climate-related mortality per 100,000 people was calculated as the sum of two mortality causes that are commonly regarded as related to climate change and climate events. Mortality rates related to exposure to high temperatures are higher in the northern parts of Sub-Saharan Africa, Saudi Arabia, and Southeast Asia (a). Mortality rates related to Malaria are concentrated especially in the northern part of Sub-Saharan Africa (b).
